# The Morbid Effects of Decayed Teeth and a Diseased Mouth upon the Health of the General System

**Published:** 1845-03

**Authors:** Cyrenius O. Cone

**Affiliations:** Barnstable, Mass.


### ARTICLE IV.

*The Morbid Effects of Decayed Teeth and a Diseased Mouth upon the Health of the General System.* By CYRENIUS O. CONE, D. D. S., of Barnstable, Mass.

IN writing upon this subject, at this time, I do not propose to enter as fully into its consideration as its merits would permit, and, perhaps, demand.

It will not be necessary in this dissertation, to enter minutely into the anatomical structure of the mouth, but merely to say, that the mouth is an oval cavity; the first of the digestive tube, which is bounded, superiorily, by the palate and superior maxillary bones; inferiorily, by the tongue and buccal membrane which covers the inferior maxillary; anteriorily, by the lips and their aperture, the mouth proper; posteriorily, by the palate and the next portion of the tube, with which it is connected, the pharynx; and, laterally, by the muscles which form the cheek. It is invested, or lined, by a mucous membrane, which is largely supplied with follicles, and into it the ducts from the different salivary glands pour their secretions. Each of the jaws, or maxillary bones, "has a prominent border, forming a semicircle, in which the teeth are implanted. This border is called the alveolar arch. The teeth are small organs, of density superior to bone, and covered, externally, by a hard substance called enamel." The teeth are supplied with blood from arteries, given off from the internal maxillary of the external carotid artery. Their nerves are from the fifth pair, which nerve, according to Sir Charles Bell, is composed of two portions, a sensitive and a motor portion. The fifth pair of nerves are divided into the opthalmic, the superior and inferior maxillary nerves, and these again are subdivided into innumerable branches. By the superior maxillary nerve, or one of its deep seated branches, the teeth are in union with the great sympathetic nerve, and, consequently, connected with all the vital organs of the body, which cannot but be considered highly important, in a pathological point of view. The other parts of the mouth, to which reference has been made, are nourished by blood from arteries which are derived from the external carotid, and the nerves which are distributed to the parts, are from the fifth, seventh, eighth and ninth pairs.

In this dissertation, I shall endeavor to confine myself strictly to the text, of "the morbid effects of decayed teeth, and a diseased mouth, upon the health of the general system," and shall avoid discussing, at this time, the effects of other diseased parts of the body, upon this cavity and its appendages.

It requires but a short period of study of human anatomy, to impress one with wonder and admiration, for the completeness of his organization. Every part is peculiarly adapted to subserve the purpose of its design. Who can contemplate the mechanical arrangement of the forearm, the construction of which gives the greatest possible degree of motion; or the structure of the lungs, one of the designs of which is to present the largest possible surface in the smallest space; without internally exclaiming, "we are fearfully and wonderfully made?" Each of these respective parts, which are so perfect in themselves, are connected by links of an unending chain; the nervous system, which is not less perfect in its construction or arrangement. For an illustration of this nervous connection, we have only to refer to the familiar cases of deranged digestion, producing pain of the head and lassitude; pregnancy, producing nausea, or pains of the head and teeth; a puncture of the foot, lockjaw; stone, of the bladder, numbness of the thigh, or pain and swollen testicle, or the action of a particle of snuff or other irritating substance impinging upon the Schneiderian membrane, in producing a powerful effort of the whole respiratory apparatus.

This action of one organ upon another at a distance, or in close contact, is what is called sympathy. As we have the word occurring for the first time in this paper, it may not be amiss to say a few words respecting it. We need not employ much time in explaining the term sympathy, as that has been already done, by the foregoing illustrations. But the term sympathy, is sometimes employed to "express our ignorance of an agent, or its mode of action, nearly as we apply the epithet vital to a process which we are incapable of explaining, by any physical facts or arguments."* The manner by which one organ sympathises with another is various. "In many cases the impression made upon the parts is carried to the brain, then reflected to the sympathising organs." As the sharp and lacerated edge of the alveolus, after the extraction of a tooth, has been known to cause great pain, and even temporary paralysis of the arm and hand, "and this is probably the way in which medical agents produce their effects through sympathy."† Or one organ may produce an effect or influence on another in health, or disease, through what is termed "sympathy of continuity," this is such as occurs between various parts of the body and membranes, which are continuous, or of like structure. For instance, the slightest taste of a nauseous substance may bring on an effort to vomit; the whole of the first passage, being unfavorable for its reception. In disease we have many examples. During dentition, the child is subject to various gastric and intestinal affections. If a source of irritation exists in any part of the intestinal, or other mucous membrane, no uneasy sensation may be experienced in the seat of the irritation ; yet it will be felt at the commencement, or where it commingles with the skin. Itching of the nose may indicate disease of the digestive mucous membrane; itching or pain of the glans penis, indicate stone of the bladder, and every dentist, who has practice and observation, has had this mode of sympathy illustrated. By the application of artificial teeth, some irritation might be set up, and the patient will complain of soreness, and pain in the pharynx; or when the introduction of teeth excites the salivary glands to pour their secretions to an unnatural extent into the mouth.

There is another variety, differing somewhat from the above, and is familiar to every pathologist. I now speak of that sym-

* Dunglison's Human Physiology.

†Dunglison's Therapeutics and Materia Medica.

pathy which exists between organs of like structure and function. "This is seen when the feet are exposed to cold and moisture, derangement takes place in the capillaries of the feet, and this derangement is reflected to every part of the capillary surface, so that in a dozen individuals exposed to this cause of disease, the derangement may be as variable in their seat as the individuals themselves." We have examples of this sympathy in the metastasis of acute rheumatism, from the fibrous structure of the joints, to the pericardium ; in every exanthematicous disease, when the danger is more or less dependant upon the degree of the affection of the mucous membrane ; or, in warm climates, where the direct rays of the sun, beaming upon the capillary surface, will induce diarrhœa or dysentery.

There is still another road by which sympathy travels from one organ to another. I now refer to that sympathy, where the primary cause is located, or the irritation is made, upon one branch of a nerve, and the sympathetic pain is felt, or reflected, and located in another branch of the same nerve, or where the impression is made upon the trunk of a nerve, and the impression is felt at another part of the same trunk, or at the extremity of the same nerve. We have illustrations of this in the disease of the heart, which will give rise to pain in, and running down the left arm. In disease or irritation of the liver, pains will be felt in the shoulder. "Thus, filaments of the phrenic nerve, penetrate the diaphragm, and communicate with the ganglia that lies around the Cæliac artery, other filaments are distributed to some of the muscles about the shoulder." "The irritation in these cases is applied to one branch of a nerve, and reflected to another branch of the same." Stricture, or irritation of the urethra, produce pain in the feet. We have illustrations of pain in the trunk, or the extremity of a nerve, by reference to some cases, which are related by Dr. Thomas Watson, in his Lectures upon the Principles and Practice of Physic, a few of which I will copy. "A man was admitted into the St. George's Hospital, on account of severe pain on the inner side of the knee-joint. The joint was carefully examined, but no marks of disease could be detected in it. On tracing the limb upwards, however, an aneurism of the femoral artery was discovered, as big as an orange, in the thigh. This the patient thought nothing of; his only concern was the pain in his knee. Sir E. Home performed the usual operation for aneurism, and the moment the ligature was drawn firmly around the artery in the upper part of the thigh, the tumor ceased to pulsate, and the pain in the knee ceased also. This man died four or five days after the operation, and on inspection of the limb after his death, the aneurism was found reduced one-half its former size, and some branches of the anterior crural nerve, which passed over it, and must have been kept upon the stretch previously to the operation, were seen to terminate in the part to which the pain had been referred on the inside of the knee." Another similar case is spoken of by the same author, who refers the case to the British Medical and Chirurgical Transactions.

"A sailor was wounded by a musket-ball in the arm. The wound healed, but the patient remained affected with agonizing pain, beginning in the extremity of the thumb and fingers, except the little finger, and extending up the forearm. His sufferings were so great that he willingly submitted to the amputation of the limb, and the operation gave him complete relief. When the amputated limb was dissected, a small portion of lead which seemed to have been detached from the ball, when it struck against the bone, was found imbedded in the fibres of the median nerve." Should any one have the curiosity, he might give himself a practical lesson upon this part of my subject by striking the elbow a little on the inside, by which operation he will compress the ulnar nerve, and a sharp thrilling sensation will be felt in the end of the little finger, and extending down the hand at the extremity of the nerve. I have, necessarily, prolonged this branch of my subject, much beyond my wish. But before speaking of those diseases of the general system, which the teeth and a diseased mouth gives rise to, or stimulates to action; I will stop to put the following query.

If facts above stated, prove what may be done by a thousand other facts, that remote organs are connected by the laws of sympathy, and exert an influence upon each other during health and disease, can it be placing the teeth and mouth in too prominent a situation in the animal economy, when we assert, that when they become diseased, other parts of the system suffer a corresponding degree of decay or derangement?

Having thus imperfectly illustrated sympathy, and some of the roads by which it travels through the system, we are prepared to direct our attention to the morbid effects of the teeth and mouth, upon the general system when diseased.

When the teeth are diseased, they may produce either local or general, or, in other words, constitutional disease. The local affections caused by diseased teeth, gums and their sockets, are sympathetic pain in the face. Neuralgic pains; inflammation of the eyes; destruction of the cellular tissue between the cheek; pain in the ears, side of the head, defective smell and taste, and disease of the jaw-bone. Most of this is easily explained by a reference to what has before been said respecting sympathy, and the nervous connection of the parts. That the teeth cause much constitutional disturbance, both in health and disease, is beyond question, and it is a subject which has attracted the attention, I may say, of all dental, and many medical and surgical writers. The justly celebrated physiologist and anatomist, Dr. John Hunter says, "The preservation of the teeth and mouth is of the utmost importance, not only as organs useful to the body, but on account of other parts with which they are connected, for disease of the teeth is apt to produce disease of the neighboring parts, frequently of a very serious consequence."

"One might at first imagine, that the diseases of the teeth are very simple, and like those which take place every where else in the bony parts of our body, but experience shows the contrary. The teeth being singular in their structure, have diseases peculiar to themselves. These diseases, considered abstractly, are very simple, but by the relations which they bear to the body in general, and to the parts with which they are immediately connected, they become extremely complicated. The diseases which may arise in consequence of those of the teeth are various, such as abscesses, carious jaw bones," &c.*

The great American writer and physician, Dr. Rush, speaks of the subject thus:

"When we consider how often the teeth when decayed, are

*Hunter, p. 132.

exposed to irritation from hot and cold drinks, and aliments; from pressure, by mortification, and from the cold air, and how intimate the connection of the teeth is with the whole system, I am disposed to believe they are often unsuspected causes of general, and, particularly, of nervous diseases;" and the same author goes a little further on, and says, "It is not necessary that they, (the teeth,) should be attended with pain in order to produce disease, for splinters, tumors, and other irritants, before mentioned, often bring on disease and death, when they give no pain, and are the unsuspected cause of them."*

One of the most frequent results of decayed teeth, is inflammation and alveolar abscesses. These abscesses are not confined, as their terms would seem to imply, or their effects are not, to the alveolar processes, but they often extend themselves, and end in caries and necrosis, and consequent exfoliation, and fistulous abscesses of the bone and jaw.

"The susceptibility and tendency of the organism, or part upon which they act, determines their character, and this is true, both with regard to constitutional and local disease of the physical structure generally, and all its parts, separately considered, but none more so, than the teeth and gums."† A number of cases in illustration of the extensive destructive effects of alveolar abscesses, which are the product of decayed teeth, has come under my notice, one of which I will mention. A gentleman having had the nerve of the third left inferior *molaris* exposed by caries, was attacked, with what, in the popular jargon of the day, is termed "ague." He applied to a dentist for the extraction of the tooth at the time, and when the face was much inflamed and swollen. The dentist, in the effort to extract, broke off the crown of the tooth, and after several ineffectual efforts to extract it, he informed the patient that it was utterly impossible, let the consequence be what it might, to remove the tooth, as it was what he termed "locked." The gentleman returned home, feeling that there was no other alternative, but to submit to the affliction, and after five months of the most agonizing suffering, during which period he was often tempted by

* Rush's Med. Enquiries, vol. 2, p. 201.

† Harris' Chars. of Teeth and Gums.

the severity of his pains to put an unnatural end to his existence, to free himself from his sufferings; he was, in some degree relieved by an abscess, opening a little anterior, and below the ear, near the angle of the inferior maxillary. After a period of nine months from the attempt to extract the tooth, with the abscess as described, he became my patient. I, much to the gentleman's astonishment and ease, extracted the fangs of the tooth, and after dissecting away a portion of the diseased bone, and bringing the wound or edges of the opening together, and with proper dressings and cleanliness of the parts, the sinus healed, and the patient was restored to perfect health. Other cases might be cited in illustration, but I will enumerate but one more.

"Mrs. K. of Philadelphia, a lady about twenty-eight years of age, had been suffering for some time from an abscess under the chin of the left side. Being ignorant of the nature of the disease, she sought no surgical aid, under the hope it might get well of itself, but it grew worse, and was attended with a constant discharge of fetid pus, with great pain in the whole jaw and mouth, extending as far as the ear. These symptoms at last became so violent as to produce general disease and fever. At this time Dr. Physic was consulted, who having discovered the malady to be a fistulous abscess passing through the under maxillary cavity and bone, immediately suspected that the teeth were the cause of it, and desired that I should be consulted. On examination, I found that three large grinders, on the side affected, had been so completely carried away by decay, that the roots remained entirely covered by the gum, which was greatly inflamed.

"1819, Oct. 8. These roots, six in number, were extracted and so effectual was the relief obtained from this operation, that in a very short time the patient perfectly recovered.

"Oct. 19. Eleven days afterwards, some chronic pains were felt in the opposite side of the jaw, and, on examination, the chin was found indurated and swollen in some degree, and the points of two roots of the second large grinder being completely covered by the gum, and immediately behind the first large grinder, were detected with great difficulty by the probe, I proposed the immediate removal of these roots, having no doubt that a new disease was forming on that side of the same nature as that of the other side. The operation was readily acceded to, and successfully performed in a few minutes, notwithstanding the great difficulty inseparable from their situation, and the parts were restored to health in a short time."

"By this early and decided treatment, the otherwise unavoidable exfoliation of the maxillary bone, which had, indeed, occasioned a great defect of substance, and a considerable scar on the left side of the chin, was entirely prevented on the right."*

We will here remark, that alveolar abscesses often develope themselves by the irritation of the temporary teeth, before the moulting of the permanent teeth, at which time the system is, perhaps, more susceptible to irritation than at a more adult age. I have often seen extensive ulceration of the buccal membrane, and muscles of the cheek; sub-acute inflammation of the pharynx, to say nothing of the destructive influence exerted upon the permanent teeth, by this source of irritation, to make it apparent to every one, that the temporary teeth demand watchful attention. The same general effects may be produced at one age as at another.

That the various diseases incident to the maxillary sinus, are, properly, more frequently referred to secondary irritation, propagated by diseased teeth and mouth, I think will be generally admitted, and for a more extensive discussion of that interesting subject, than this paper will permit, I will refer to Professor Harris' work upon this subject, and papers upon the same disease, to be found in the American Journal of Dental Science by Drs. Hullihen and Thackston.

A constitution which is predisposed to osteo-sarcomatous, cancerous and other tumors, may have them developed by dental irritation. That preternatural and spongy gums, fungous and cartilaginous growths of the mouth, are more frequently symptomatic than idiopathic, and these sympathies claim, in most cases, dental irritation as their parent, we have proof of in many dental works, where the teeth have been extracted, and

* Koecker's Dental Surgery.

by this means the primary cause of the disease removed, and the cures have been complete.

One case which I was requested to see by a physician, who was a friend of mine, and in attendance, I will relate. The patient was a married lady, twenty-eight or thirty years of age; of a scorbutic habit; had been confined to her room, and most of the time to her bed, for two years. Her medical attendant, to whom reference has been made, had but recently been called in, but he at once suspected that her illness consisted, or was dependent upon the unhealthy state of her mouth.

On examining the mouth, I found the gums very much ulcerated and swollen, so as to cover two-thirds of the crowns of those teeth which were not completely destroyed by decay. The whole of the pharynx, as far as the eye could reach, was highly inflamed, and the discharge of fetid matter into the mouth was such as to render the air at any part of the room intolerable, and the tongue was red upon its edge and covered with a thick coating, and when requested to extend it from the mouth, it was extended with an effort, or, in other words, it was first extended a short way, and then, as if from diffidence, suddenly withdrawn, and then, apparently, by a great effort it was thrust from the mouth where it was retained. We extracted for this patient, at different periods, all decayed teeth and stumps, and such other teeth as had lost their vitality or connection with the neighboring parts, and with such remedies as were addressed to the constitution, which, principally, consisted of the different preparations of iodine, mingled with tonics, the patient's general health improved, and the mouth lost the fungous growth, and the ulcerated and inflamed parts assumed a healthy character, and in about six months the patient was able to attend to the lighter duties of her family.

*Ozæna*, a painful and peculiarly unpleasant disease, oftentimes claims a diseased denture as its causes. A highly interesting and instructive case is related of this disease, which was caused by a diseased tooth, and cured by its removal, by Professor Harris, in the Maryland Medical and Surgical Journal, and was copied into the American Journal of Dental Science. Its length will not permit of its insertion in this paper.

*Neuralgia* may be added to our catalogue of diseases which oftentimes is indebted to dental irritation, for its painful acquaintance with the afflicted. This disease usually exists as a constitutional disease, and often acknowledges the same cause as ague, namely, the miasma of marshes or *malaria*, but its slumbering ashes are often fanned into life, and to the painful exhibition of its presence, by a diseased mouth. Mr. Bell, who has given the subject a good deal of attention, divides the disease into that which is dependent upon constitutional derangement, and such as are dependent upon local causes. Mr. Bell gives a number of highly interesting cases in illustration, but as their length may be an objection, and justice could hardly be done them by division, I shall venture to substitute a case that came under my notice, and now occurs to my mind. A chlorotic young lady presented herself to me for advice. She complained of great pain, commencing in or about the ear, which would be reflected upon the side of the head, down the arm to the wrist, where it would locate for a while, then be found in the left side, in the neighborhood of the stomach, and again, at times, it would commence near the ear, and follow the course of the inferior maxillary nerve, and stop suddenly at the symphisis of the jaw. I examined attentively the mouth and teeth, and found an inferior molar tooth, which had before been treated with some substance which had destroyed its vitality, and was applied under the title of "nerve paste," and afterwards plugged.

This tooth, upon striking it a slight blow with an instrument, gave rise to all the symptoms above described. I at once informed her that she could not expect relief until extraction was performed. She immediately assented, and the operation was satisfactorily performed, since which time she has been entirely free from the pains of the face, head, &c. I omitted to say, that the above young lady had been treated with steel and other tonics. Mr. Descot mentions a case of neuralgia, of ten years standing, which was relieved by the extraction of a carious tooth.

*Hemicrania* is a disease which is a near kin to neuralgia. "Hemicrania is simple head-ache, confined to one side, and generally occupying the brow and forehead, but sometimes affecting, very extensively, one moiety of the head."* Dr. Darwin observes of this disease, "when this affection is confined to a definite point of the parietal bone, or on one side, it is generally termed *clavis hystericus*, and is always I believe owing to a diseased *dens molaris*."

*Epilepsy* is another of the nervous diseases which are greatly influenced by the teeth, both while in health and in disease. It is not within our province, or design, to enter into a pathological description of this disease, and will merely say, that it may be caused by any impression upon the great nervous centre. It has been observed by some one, "that if you can trace the early history of an adult epileptic, you will most always find that he, or she, suffered infantile convulsions," and these convulsions are, in seven times out of ten, the result of dental irritation. "Then follows the trying period of teething. In a few years more, second dentition occurs," and it will be seen that anterior to the period of second dentition the majority of epileptic patients are attacked. Of sixty-six epileptic women, noticed by Dr. Watson, thirty-eight were attacked previous to puberty, at about the age when second dentition is being completed. In Dr. Rush's Medical Enquiries, vol. 1, we find a case which was dependant upon decayed teeth, and was cured upon their removal.

"Sometime in the year 1801, I was consulted by the father of a young gentleman of Baltimore, who had been afflicted with epilepsy. I inquired into the state of his teeth, and was informed that several of them, in the upper jaw, were decayed. I directed them to be extracted, and advised him afterwards to lose a few ounces of blood, any time he felt the premonitory symptoms of a recurrence of his fits. He followed my advice, in consequence of which, I had lately the pleasure of hearing, from his brother, that he was perfectly well."

I will also relate a case told me by a medical friend, which occurred in his practice. One of his patients had been suffering from a tooth-ache, and, while travelling, he was seized with a slight epileptic attack. Upon arriving at the first village, he applied to a physician, who prescribed him some effervescent draught, and told him to continue his journey home. Some two

* Watson's Practice of Physic or three days after this, when at home, he was attacked with another fit, which was more severe than the one before, and was followed by some chronic pains in the tooth that had been painful. This drew the attention of the medical attendant in that direction, which resulted in the removal of the tooth and pain, and the recurrence of the epileptic attacks.

Dental disease has its influence in the production of *phrenitis*. A very illustrative case is given in the American Journal of Dental Science, vol. 2, page 141, by Dr. J. E. Snodgrass. The subject of this fatal case was Dr. S's. father. The case is highly interesting and instructive, but its length will not permit of a further notice.

*Mania* is also recorded among the diseases and unhappy effects of a diseased denture. A case of which is related by Dr. Rush, in his Medical Enquiries upon the disease of the mind, where the mania was produced by teeth which were not painful, but, probably, had lost their vitality. A case of mania, from decayed teeth, is related in the American Journal Medical Science, and copied into the American Journal Dental Science, by Dr. Wm. Mendenhall, of N. C. The case is related by him thus:

"A black boy, about twelve or fourteen years of age, was attacked with mania in the month of May, 1840, supposed to have arisen from over-heating, in trying to subdue a fire, which broke out upon his owner's fencing. Cathartics, blistering, and cupping, and venesection, with many other remedies were tried, which relieved him in some measure, during a great part of the year 1841. But in January, 1842, he became quite a maniac again, and continued so, notwithstanding all of the above remedies were used, together with shaving his head, and the application of cold water, and the opening of the temporal artery, and the loss of considerable blood. A few weeks ago it was discovered that he had two decayed teeth in the upper jaw, one on the right, the canine tooth, and the other on the left, one of the anterior molars, both of which were extracted. Since which time he has been quite restored. The day before the extraction, he escaped from the family, and passed the night in the woods, and was caught in the act of running away, after coming near or to the house in the absence of the family. Since that time he has regularly attended to business, without showing any signs of insanity."

Dyspeptic conditions may arise from disordered teeth, as dental disorder may arise from dyspepsia. Sympathies cannot be one-sided, and as the teeth suffer from a disordered stomach, so the stomach will suffer from disordered teeth.* The stomach is endowed with a remarkable degree of sympathy. In fatal cases of gastritis, death (as Bichat describes) begins at the heart. A sudden blow upon the epigastrium, may put a stop to the movements of the heart, and result in fatal syncope, without leaving any local traces of its operation. On the other hand, a person in an extreme state of exhaustion and faintness, will, sometimes, rally upon swallowing a number of ounces of brandy into the stomach, and recover his pulse and color much too soon, to ascribe the cause or effect to the absorption of the alcohol into the circulation.† It would not appear necessary that the cause of irritation should be of an extensive character. The sharp and irritating edge of a decayed and broken tooth, may cause disease of the digestive organs. "A sharp pointed feather fixed in a person's clothes, in such a manner as to goad him constantly in some tender or irritable part, will produce not only a wasting of flesh, but a real dyspepsia."‡ Again, the intense pain caused by a diseased denture, will engender symptomatic fever, which will connect itself with digestion, and entail upon the system countless evils. It is a well ascertained fact in pathology, that acute pain in any part of the body will frequently affect many of its functions, through sympathy, as I have before explained, and as the stomach is the great centre of nervous connection, it is peculiarly susceptible to dental or other irritation. And digestion and assimilation may be but partially or imperfectly performed, by painful and diseased teeth, calling away from the stomach that nervous energy which is necessary for perfect and healthy digestion, to the painful parts. Healthy teeth have justly been compared to grave-stones, indicating the quiet, effectual and peaceful resting place of all foods, which enter the

*American Journal Dental Science, vol. 4, p. 29.

† Watson's Principles and Practice of Physic.

‡ American Journal Dental Science, vol. 1, p. 267.

digestive portals. But when the teeth are decayed and lost, and do not perform the functions properly for which they were designed, it throws a double duty upon other organs, which will sooner or later yield to disease, and then our food, instead of finding a peaceful and quiet resting place, call with the impatience of St. Patrick's snake, but in *painful* language, for a more decent burial.

I need not employ any time in proving that the teeth, when diseased, soon implicate in disease the neighboring parts, after what has been said under the head of alveolar abscesses, but will simply add, that when other parts of the mouth become diseased, the healthy secretions of the cavity become vitiated and rendered unfit for the office they perform in digestion; and not only render digestion imperfect, but in the process of assimilation, convey disease even into the circulation.

I shall not deem it best to introduce any cases in illustration of the above, from a number, which are highly interesting, that are before me, from the increased length that it would give this paper.

*Deafness* has been caused, and completely relieved by proper attention to the restoration of the health of the mouth. And this is not at all surprising when we consider the connection which exists between the parts, by the nervous union of the *chorda tympani* with the fifth pair, or the direct communication which exists between the mouth and the delicate apparatus of the ear, through the channel of communication, the eustachian tube. By this communication, an inflamed mouth, caused by diseased gums, alveolar processes, or teeth, may extend itself to the ear, as is often illustrated by malignant cases of scarlet fever. A cure is related in the third volume of the American Journal of Dental Science, by Dr. Koecker, by which a case of perfect deafness was completely cured by restoring a diseased mouth to health.

Disease of the eye, and loss of sight, is often connected with dental irritation and a diseased mouth. We have cases from the best authorities, of recovery from various affections of the eyes, by proper directed attention to the mouth. I copy the following from Dr. Watson's Principles and Practice of Physic. In speaking of amaurosis, he says, "It has obscure connections with teeth, probably through irritation of the facial branch of the fifth pair."

"A physician of my acquaintance, residing in London, has a young son, who, on a number of occasions, caused him great uneasiness, by becoming blind in one eye, without any obvious cause, and without any visible change in the organ, but the blindness on each occasion had gone off again, apparently in consequence of the extraction of some teeth which were growing irregular."

"I am assured by Dr. Ashburner, that such cases are common." Mr. Lawrence relates a very singular case of dental irritation, giving rise to amaurosis. "A man, thirty years old, was suddenly attacked with violent pain in the temple and eye, then that side of the face generally. The pain continued to recur, from time to time, until, at length, he discovered he was blind in the left eye. By-and-by the cheek swelled, and some spoonsful of bloody matter were discharged by a spontaneous opening in the lower eye-lid, and then the pain subsided, but, after some months, returned with great severity. The patient then went to Wilna, with the view of having the eye extirpated, and consulted Professor Galenzonski, who found the eye totally insensible to light, with the pupil dilated, but no other visible alteration. He ascertained, however, that the first molar tooth on that side was carious; it had never caused the patient much uneasiness, and the tooth-ache, which he had occasionally suffered, had not been coincident, in point of time, with the pain in the head or eye. Dr. Galenzonski thought proper to extract this tooth, and was greatly surprised at seeing a small substance protruding from the extremity of the fang. This proved to be a little splint of wood, about three lines in length, which had perforated the centre of the tooth, and had, probably, been introduced in using a wooden tooth-pick. A probe passed from the socket into the antrum, from which a few drops of a thin purulent fluid escaped. The pain ceased almost entirely, and, on the same evening, the eye began to be sensible to light. The vision gradually improved, and on the ninth day from that time, after thirteen months' blindness in that eye, he was able to see with it as perfectly as with the other."

I proceed in the next place to speak and record amongst those diseases which are greatly influenced by diseased condition of the teeth and mouth, that fatal and almost hopeless disease, which runs riot among the young, the most gifted and beautiful of the human race, and is known to every one under the titles of pulmonary consumption, or, *tubercular phthisis.* The irritation and constitutional derangement of first dentition, often sow the seeds of this disease, which are to be cherished and brought to maturity by subsequent disease of the mouth. It would not require much effort to pursue, step by step, how inflammation might travel down the soft parts of the mouth, to the delicate substance of the lungs. And I will here notice, that the danger of inflammation is not in proportion to the surface that it occupies, but its location. If the fatal inflammation which terminated the life of the celebrated Washington, could have been located an inch further up or down the throat, he would not have died at that time of acute laryngitis. But the influence of a diseased mouth in producing disease of the lungs is not confined to the laws, that govern inflammation, in travelling similar structures, but the filth and disease which accompanies a diseased mouth, cannot but contaminate and poison the air which passes to and from the lungs; so that instead of performing its great and important office in the animal economy; it is freighted with the seeds of disease, which are to be taken up by absorption, into the lungs, and then mingle with the blood to be sown in every part of the frame.

I will not add to my already too long paper, cases in illustration from such as are before me, but will hasten to a close. But before doing so, I will remark, that irritation of the teeth during dentition, often produces hydrocephalus; corea, and child-crowing, and misplaced or decayed teeth may give rise to spontaneous salivation, hysteria, suspension of the catamenia, and thousands of other remote sympathies.

I would also say, that I do not intend to advocate a doctrine which would require of me to say, that these diseases are always the result of teeth and a diseased mouth, any more than I would attempt to prove, that one form of medicine would be proper for all diseases, or even for the different stages of any one disease. But the conclusion to which I would lead, is, that the teeth and mouth are important parts of the system, and that when diseased, vital organs may be implicated, and that the diseases of the teeth and mouth are such, as require something more than the attention of a mere "tooth-smith," but the study and attention of skilful surgeons, who, by judgment and skill, shall be able to pluck the lurking but effective disease, when in the bud, from the system, and

> "——— wipe from beauty's cheek the tears that burn,
> And bids her rose and smile return."

